# Urodynamic investigation by telemetry in Beagle dogs: validation and effects of oral administration of current urological drugs: a pilot study

**DOI:** 10.1186/1746-6148-9-197

**Published:** 2013-10-08

**Authors:** Stéphanie Noël, Laurent Massart, Annick Hamaide

**Affiliations:** 1 Department of Companion Animal Clinical Sciences, College of Veterinary Medicine, University of Liège, 4000 Liège, Belgium; 2 Department of Animal Production - Functional Sciences, College of Veterinary Medicine, University of Liège, 4000 Liège, Belgium

**Keywords:** Urodynamics, Telemetry, Dog, Phenylpropanolamine, Oestriol, Bethanechol, Oxybutynin, duloxetine, Bladder function

## Abstract

**Background:**

Vesico-urethral function may be evaluated in humans and dogs by conventional urodynamic testing (cystometry and urethral pressure profilometry) or by electromyography. These techniques are performed under general anaesthesia in dogs. However, anaesthesia can depress bladder and urethral pressures and inhibit the micturition reflex. The primary objective of this pilot study was to evaluate the use of telemetry for urodynamic investigation in dogs. We also aimed to determine the applicability of telemetry to toxicologic studies by assessing the repeatability of telemetric recordings.

**Results:**

Conventional diuresis cystometry was performed in six continent adult female Beagle dogs prior to surgical implantation of telemetric and electromyographic devices. In the first phase of the telemetric study, continuous recordings were performed over 8 days and nights. Abdominal, intravesical and detrusor threshold pressures (Pdet th), voided volume (Vv), urethral smooth muscle electrical activity and involuntary detrusor contractions (IDC) were measured during the bladder filling phase and during micturition episodes.

Vv recorded during telemetry was significantly lower than bladder volume obtained by diuresis cystometry. Repeatability of telemetric measurements was greater for observations recorded at night. IDC frequency and Pdet th were both lower and Vv was higher at night compared to values recorded during daytime.

In the second phase of the telemetric study, phenylpropanolamine, oestriol, bethanechol, oxybutynin or duloxetine were administered orally for 15 days. For each drug, continuous recordings were performed overnight for 12 hours on days 0, 1, 8 and 15. Electromyographic urethral activity was significantly increased 8 days after oestriol or duloxetine administration. No significant changes in bladder function were observed at any time point.

**Conclusions:**

In dogs, the high repeatability of nocturnal telemetric recordings indicates that this technique could provide more informative results for urologic research. Urethral smooth muscle electrical activity appears to be modified by administration of drugs with urethral tropism. In this pilot telemetric study, bladder function was not affected by oral administration of urological drugs at their recommended clinical dosages. Experimental studies, (pharmacokinetic and pharmacodynamic) and clinical studies are warranted to further define the effects of these drugs on vesico-urethral function in dogs.

## Background

Vesico-urethral function in humans and dogs is currently investigated by conventional urodynamic testing, including cystometry and urethral pressure profilometry, and by electromyography (EMG) [1,2]. These techniques are useful to diagnose alterations in bladder and/or urethral contractility [2] but are subject to several limitations. In animals, urodynamic investigation requires general anaesthesia that can depress bladder and urethral pressures and inhibit the micturition reflex [3]. In humans, poor correlation between clinical findings and urodynamic investigations has been described [4].

Other urodynamic tests have been developed to provide more accurate data regarding lower urinary tract function. Diuresis cystometry allows for a better approximation of the physiologic bladder filling state, with high threshold volume, compliance and low threshold pressure [5]. This technique permits detection of involuntary detrusor contractions (IDC) in dogs with and without clinical signs of bladder dysfunction [6,7]. In humans, ambulatory urodynamic monitoring can be helpful as several micturition cycles may be studied in multiple physiologic conditions [8], allowing better detection of IDC compared to retrograde filling cystometry [9,10].

Urodynamic telemetry conveys significant benefits for urologic research and has been described in non-rodent species (monkeys, pigs, ovine foetuses and dogs). Urodynamic telemetry reduces stress to the animals, does not require animal handling or chemical restraint and offers a non-invasive technique for prolonged monitoring. The telemetric equipment comprises an implantable transmitter that measures pressures and biopotentials (EMG, ECG) and sends data via radio waves to a receiver. The data are then converted and analysed [11,12]. During recordings, the animals are placed in metabolic cages to measure urine production [12,13].

Telemetric recordings offer several advantages for pharmacodynamic studies. The effect of a single administration of darifenacin on bladder function in dogs has recently been investigated by telemetry [12]. However, to date there are no reports of telemetric assessment of any of the drugs currently used to treat urinary incontinence or retention in veterinary medicine.

Therefore, the aims of this pilot study were to investigate vesico-urethral function in female beagle dogs using telemetry. We aimed to measure detrusor pressure to perform EMG procedures, and also to study the effects of circadian rhythm on these parameters. Data obtained from diuresis cystometry were compared with data from urodynamic telemetric testing. The effects of oral administration of phenylpropanolamine (PPA), oestriol, bethanechol, oxybutynin and duloxetine were also investigated.

## Results

### Complications

Flank seromas were observed in 5 dogs following implantation of the telemetric device. These all resolved spontaneously within several days. Four dog developed bacterial cystitis over the course of the study. These dogs were excluded from the study whilst receiving antibiotic treatment (3 weeks) and were reintroduced following negative urine culture. In one dog, the electronic module stopped working six months after implantation due to a blood clot at the level of the connection between the pressure catheter and the electronic module. The implant was successfully removed and replaced.

### Comparison between diuresis cystometry and telemetry

The mean volume of urine recorded during diuresis cystometry was significantly greater than the mean Vv recorded during telemetric recordings (*P* = 0.027) (Table 1).

**Table 1:** Comparison of urodynamic values obtained during diuresis cystometry and during telemetry

**Cystometry**	**Pth**	**Vol**
**Diuresis cystometry**	33.7 ± 2.9	432.5 ± 77.7^a^
**Telemetry**	40.1 ± 2.4	91.9 ± 6.1^b^

Pth = Bladder threshold pressure (mmHg); Vol = Bladder volume (mL) corresponds to the volume of urine retrieved from the urinary bladder at the time of the micturition reflex during diuresis cystometry, or corresponds to the volume of urine voided during telemetric cystometry.

Data are expressed as LSMean ± SEM.

Data with different superscripts are significantly different (*P* < 0.05).

### Comparison between day and night studies

For the filling phase, urodynamic measurements were not different between day and night studies. Abdominal and bladder pressures were significantly influenced by the age, weight and activity of the dogs (*P* < 0.0001) and activity level also has a significantly influence on detrusor pressure (*P* = 0.0007). The frequency of micturition was not significantly different between day and night recordings, but significant inter-individual differences were observed (*P* < 0.0001) (Table 2).

**Table 2:** Frequency of micturition, involuntary detrusor contraction (IDC) and EMG values during day and night studies

**Parameters**	**Circadian rhythm**	
	**Day**	**Night**
**Frequency of micturition**	2.3 ± 0.2	1.8 ± 0.2
**IDC**	4.06 ± 1.14^a^	2.3 ± 1.14^b^
**EMGi/hour (mV sec)**	0.02156 ± 0.0065	0.02106 ± 0.0065
**EMGi/minute (mV sec) Pre-micturition**	0.5968 ± 0.092	0.5388 ± 0.099
**EMGi/minute (mV sec) Post-micturition**	0.5979 ± 0.092	0.5883 ± 0.092

EMGi/hour = integrated EMG during the hour around the micturition episode.

EMGi/minute = integrated EMG during the minute preceding (Pre-micturition) or following (Post-micturition) the micturition episode.

Data are expressed as LSMean ± SEM.

Data with different superscripts are significantly different (*P* < 0.05).

**Figure 1:**
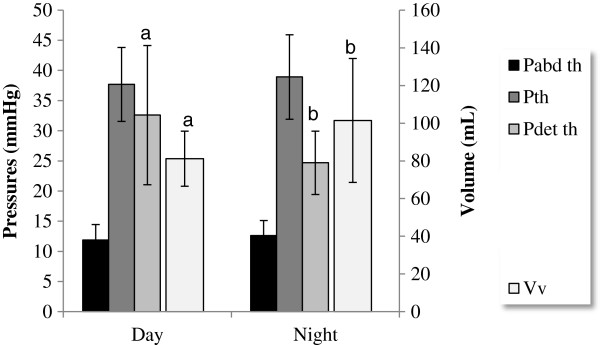
**Telemetric cystometry: comparison of urodynamic values obtained during day and night recordings (LSMean ± SEM).** Vv = voided volume; Pth = bladder threshold pressure; Pdet th = Detrusor threshold pressure; Pabd th = Abdominal threshold pressure. For each parameter, different superscripts **(a,b)** indicate significant difference (*P* < 0.05).

When considering micturition episodes, a good repeatability of Vv, Pth and Pdet th measurements as well as higher Vv values (*P* = 0.0088) were observed during night recordings (Figure 1). During day recordings, higher Pdet th values (*P* = 0.0041) and significant variability of Vv values (*P* = 0.014) were found.

IDC were recorded at a significantly higher frequency during the day (*P* = 0.032) (Table 2).

No difference in urethral electrical activity was observed between day and night recordings (Table 2).

### Drug studies

Following oestriol and duloxetine administration, EMGi/hour values were significantly increased on day 8 compared to days 0, 1 and 15 (*P* < 0.05 and *P* < 0.001 respectively) (Table 3).

**Table 3:** Urethral EMG: evolution of the EMGi/hour values during the different drug administrations

**Drugs**	**EMGi/hour (mV sec)**			
	**Day 0**	**Day 1**	**Day 8**	**Day 15**
**Phenylpropanolamine**	0.019 ± 0.004	0.015 ± 0.004	0.018 ± 0.004	0.020 ± 0.004
**Oestriol**	0.018 ± 0.004^b^	0.018 ± 0.004^b^	0.027 ± 0.004^a^	0.019 ± 0.004^b^
**Oxybutynin**	0.02 ± 0.005	0.02 ± 0.004	0.022 ± 0.005	0.016 ± 0.005
**Bethanechol**	0.016 ± 0.004	0.022 ± 0.004	0.017 ± 0.004	0.016 ± 0.004
**Duloxetine**	0.017 ± 0.004^b^	0.017 ± 0.006^b^	0.055 ± 0.005^a^	0.019 ± 0.005^b^

EMGi/hour = integrated EMG during the hour around the micturition episode.

Data are expressed as LSMean ± SEM.

Within each row, data with different superscripts are significantly different (*P* < 0.05).

During the entire course of the study, no significant effect of PPA, oestriol, oxybutynin, bethanechol and duloxetine was observed on the urodynamic parameters, the frequency of micturition or IDC, or the EMGi/minute values (Tables 4 and 5).

**Table 4:** Telemetric cystometry: evolution of detrusor pressure and voided volume during the different drug administrations

**Drugs**	**Pdet th (mm Hg)**				**Vv (mL)**			
	**Day 0**	**Day 1**	**Day 8**	**Day 15**	**Day 0**	**Day 1**	**Day 8**	**Day 15**
**Phenylpropanolamine**	31.9 ± 8.5	28.4 ± 8.2	28.2 ± 8.5	24.5 ± 9.1	101.4 ± 46.2	109.9 ± 43.2	112.2 ± 46.1	175.1 ± 50.1
**Oestriol**	22.4 ± 6.9	25.7 ± 6.9	26.1 ± 7.2	26.1 ± 7.1	139.2 ± 36.4	160.6 ± 35.9	141.19 ± 39.6	121.3 ± 38.3
**Oxybutynin**	26.6 ± 7.5	30.1 ± 6.3	25.7 ± 7.5	26.1 ± 7.3	64.9 ± 40.3	79.6 ± 31.8	55.9 ± 41.5	71.4 ± 40.2
**Bethanechol**	26.9 ± 7.9	34.7 ± 7.6	21.91 ± 7.5	24.6 ± 6.7	84.2 ± 45.4	85.8 ± 42.8	83.9 ± 43.1	129.5 ± 40.9
**Duloxetine**	30.3 ± 7.1	30.4 ± 7.9	30.1 ± 8.3	34.7 ± 7.9	119.5 ± 39.1	186.6 ± 45.6	141.7 ± 49.5	174.8 ± 45.7

Pdet th = Detrusor threshold pressure (mmHg); Vv = Bladder voided volume (mL).

Data are expressed as LSMean ± SEM.

**Table 5:** Urethral EMG: evolution of the EMGi/minute values during the different drug administrations

**Drugs**	**EMGi/minute: Pre-micturition (mV sec)**				**EMGi/minute: Post-micturition (mV sec)**			
	**Day 0**	**Day 1**	**Day 8**	**Day 15**	**Day 0**	**Day 1**	**Day 8**	**Day 15**
**Phenylpropanolamine**	0.55 ± 0.1	0.54 ± 0.1	0.82 ± 0.11	0.99 ± 0.12	0.60 ± 0.01	0.59 ± 0.1	0.62 ± 0.11	0.51 ± 0.12
**Oestriol**	0.49 ± 0.11	0.58 ± 0.1	0.81 ± 0.12	0.51 ± 0.11	0.51 ± 0.11	0.6 ± 0.11	0.64 ± 0.12	0.49 ± 0.11
**Oxybutynin**	0.57 ± 0.12	0.53 ± 0.10	0.46 ± 0.12	0.52 ± 0.11	0.50 ± 0.12	0.64 ± 0.10	0.55 ± 0.12	0.48 ± 0.11
**Bethanechol**	0.41 ± 0.12	0.62 ± 0.09	0.55 ± 0.11	0.57 ± 0.10	0.50 ± 0.12	0.67 ± 0.09	0.49 ± 0.11	0.49 ± 0.10
**Duloxetine**	0.45 ± 0.11	0.51 ± 0.11	0.54 ± 0.10	0.60 ± 0.11	0.47 ± 0.11	0.56 ± 0.11	0.49 ± 0.10	0.56 ± 0.11

EMGi/minute = integrated EMG during the minute preceding or following the micturition episode.

Data are expressed as LSMean ± SEM.

## Discussion

The results of this study demonstrate that telemetry in dogs can be a valuable tool in urologic research. Voided volumes were found to be lower when measured by telemetry, compared to measures obtained during diuresis cystometry. No change in threshold pressures was observed. In humans, the International Continence Society has defined different bladder capacities recorded during filling cystometry. The cystometric capacity is the bladder volume measured at the end of the filling cystometrogram (when the patient is given permission to void). The maximum cystometric capacity is the volume at which the patient states a strong desire to void. The maximum anaesthetic bladder capacity is the maximum volume to which the bladder can be filled under deep anaesthesia [14]. Functional bladder capacity is determined by the use of a voiding diary [2]. We believe that data obtained by telemetry in dogs may be used to derive values for functional bladder capacity or maximum cystometric capacity, while volumes obtained during diuresis cystometry could represent maximum anaesthetic bladder capacity. During conventional cystometry, the bladder is filled in a non-physiological way leading to variability especially if high filling rates are used [15]. High filling rate can be associated with mechanical trauma to receptors, nerve endings and cell junctions. Bladder wall damage may alter the frequency of action potentials and provoke a decreased desire to void until larger bladder volumes are reached [5,16]. Although diuresis cystometry has been described as a more “physiological” way to investigate bladder function in dogs, it remains an artificial filling method, altering urodynamics.

Repeatability was good for measurements obtained during the night emphasizing the importance of physiological circadian variations on urodynamic investigation. In an earlier 24-hour telemetry study in dogs, McCafferty et al. (2009) [12] described differences between bladder pressure in the dark compared to hours of light. Bladder pressure also varied during periods of enhanced locomotor activity.

In the present study, in addition to the routinely reported urodynamic parameters, detrusor pressure was also investigated as it provides a more accurate evaluation of bladder function. Detrusor pressure represents the component of intravesical pressure created by active and passive forces in the bladder wall without the influence of pressure from other abdominal sources [14]. In this study detrusor pressure values were higher during daytime than at night.

Voided volumes were also increased at night, supporting previously reported results in rats and humans [17-19]. Circadian variations may not be solely dependent on the diurnal variation in glomerular filtration rate and urine production, but could also depend on a neural or bladder-based mechanism that contributes to variations in bladder capacity [17]. In this study, no difference in micturition frequency was observed between days and nights. These findings contradict previous results in experimental animals and humans. Increased urination frequency during periods of dark has been described in rats [17] and a higher micturition frequency has been described during the day in healthy human beings [15]. Furosemide has been used during a 24-hour telemetric study to increase micturition frequency [12]. However, while furosemide increased voiding frequency and volume per void, it caused a decrease in the peak micturition pressure [12]. We chose not to use furosemide in the present study to limit interference with physiological values. This approach may have contributed to the lack of variation in micturition frequency between nights and days.

IDC, without urine loss, were more frequently observed during the day than during the night and were not observed during diuresis cystometry. IDC frequency was not affected by the administration of any of the drugs tested. Detrusor instability/idiopathic detrusor overactivity is characterized by involuntary detrusor contraction during the filling phase, may be spontaneous or provoked and lacks a defined cause [14]. Compared to human studies, the frequency of IDC here was lower, probably because higher cut-off pressure values were chosen. In a previous telemetric study in pigs, IDC were only observed in pigs with partial bladder outlet obstruction [11]. The clinical relevance of IDC in our healthy experimental dogs is questionable. IDC could reflect physiological detrusor activity not detectable during conventional filling cystometry due to bladder desensitisation [20]. Alternatively, IDC could reflect contact between the pressure sensor and the bladder wall [21]. The higher frequency of IDC observed during the day may reflect higher daytime activity or differences in the dog’s positioning. Greater detrusor activity is detected in humans if cystometry is performed in a standing or sitting position [22].

To the authors’ knowledge, urethral smooth muscle electrical activity has not previously been investigated using needle electrodes in dogs. In female dogs, the smooth urethral muscle is the preferred therapeutic target for the medical treatment of urethral sphincter mechanism incompetence (USMI) [23,24]. Despite the known contribution of smooth muscle to the maintenance of resting urethral tone, we did not observe any variation in urethral smooth muscle electrical activity during the first phase of the study. It is possible that the contribution of urethral smooth muscle to resting urethral tone induces insufficient electrical activity to activate the EMG equipment. Smooth muscle fibres are arranged in a syncytium with poorly defined synapses. The summation of extracellular currents in smooth muscle is more random with a smaller net potential, preventing the formation of reproducible motor unit potentials [25]. Smooth muscle action potentials are calcium ion dependent, while those in striated muscle depend on transmembrane sodium ion currents. The extracellular current in smooth muscle may be smaller because of the relatively lower membrane expression of calcium channels compared to sodium channel expression in striated muscle [26]. In women and rats, urethral smooth muscle activity has been recorded using surface or needle electrodes [25,27]. Needle electrodes are influenced by the electrical activity within a mean distance from the tip of 0.5 mm allowing the recording of a single motor unit whereas surface electrodes provide a composite picture of muscle activity [28].

PPA stimulates α-adrenergic receptors in the smooth muscle of the bladder neck and proximal urethra by acting as an indirect sympathomimetic amine [29-31]. It also stimulates β_1_- and β_2_-adrenergic receptors by inducing the release or inhibiting the reuptake of norepinephrine [32-34]. In this study, no change in urethral smooth muscle electrical activity was observed following oral PPA administration. This finding contradicts previous work showing that PPA is effective in improving urethral function in continent and incontinent bitches [35-39]. After two weeks of PPA administration, a clear trend towards decreased detrusor threshold pressure and increased voided volume was observed. This finding matches urodynamic changes observed previously in response to a single oral administration of PPA at this dose [40]. Overall, results from this study, though not statistically significant, support the hypothesis that PPA induces bladder relaxation by acting on β-adrenergic receptors in the bladder wall [41].

Various reviews in human medicine have summarized the effects of oestrogens on the lower urinary tract and their efficacy in the treatment of stress urinary incontinence (SUI) in women [42-44] and USMI in bitches [45,46]. However, evidence of clinical efficacy is lacking. Oestrogens increase the number and responsiveness of α-adrenergic receptors to sympathetic stimulation [25,47], and increase blood flow to the urethral tissues [48]. They stimulate cellular proliferation and improve the maturation index of urethral epithelium [49]. Oestrogens may act on β_3_-adrenergic receptors [50] and on muscarinic receptors though this remains unproven [47,51,52]. In the present study, the electrical activity of urethral smooth muscle was significantly increased after 8 days of oestriol oral administration. This finding supports a previous urodynamic study on anaesthetized dogs that reported a significant increase in urethral pressure after 7 days of daily administration of oestriol [46]. A stimulating effect of oestrogens on the urethral smooth muscle may therefore be suspected.

Bethanechol is a parasympathicomimetic drug indicated for the treatment of urinary retention. However, results of experimental studies in mice, cats and dogs are inconclusive [53-55] and evaluations of its clinical efficacy in humans and dogs have been disappointing [56-59]. The effects of bethanechol on bladder function have not yet been thoroughly assessed in veterinary medicine. In this study, 15 days of oral administration of bethanechol at recommended clinical dosages caused no change in urethral or bladder function. Further studies are required to investigate the potential effect of bethanechol at different dosages on vesico-urethral function.

Oxybutynin is a muscarinic receptor antagonist with effects on the detrusor muscle [60]. It is commonly used in human patients for the treatment of detrusor instability [61-65] and its use has been reported in a dog and a cat [66]. Previous experimental studies in rat and dog models demonstrated an increased bladder volume with decreased bladder contraction pressure after high doses of oxybutynin [67], or an increased bladder volume without variation of bladder pressure [68,69].

In this study, we did not observe any changes in bladder function following oral administration of oxybutynin at dosages extrapolated from human literature. McCafferty et al. (2009) recently reported the effect of a single administration of darifenacin, a M_3_ receptor selective antagonist, on telemetric urodynamics in dogs. The described decrease in peak micturition pressure could also be attributed to the administration of furosemide during the experiment [12].

Duloxetine is a balanced serotonin-norepinephrine reuptake inhibitor acting at the presynaptic neurons in Onuf’s nucleus of the sacral spinal cord [70,71]. Though duloxetine is commonly used in women with SUI [70,71], reports of its use in dogs with bladder or urethral dysfunction are rare. Duloxetine amplifies the effect of glutamate in Onuf’s nucleus, increasing pudendal nerve activity. Duloxetine also plays a role in initiating urethral striated muscle contraction during bladder filling [72,73]. In women with SUI, the clinical efficacy of duloxetine is based on stronger urethral contraction and persistent sphincter tone during the storage phase [74,75]. In this study, after 8 days of oral administration of duloxetine, an increase in urethral smooth muscle electrical activity was recorded, while no change in bladder function was observed. EMG studies of urethral smooth and striated muscle are warranted in dogs to further investigate the potential effects of duloxetine and its viability for the treatment of dogs with refractory urinary incontinence.

The principal limitation of this pilot study is the small number of dogs included. We tried to compensate for this by using the telemetry tool to record multiple micturition events per dog thereby maximising available data. Another important limitation is the lack of a defined dose for each drug; due to financial restrictions in this study, evaluations of the drugs at a range of different doses was not performed. With the exception of PPA [35], no studies have been published comparing the pharmacokinetic and pharmacodynamic properties of these drugs. The dosages were chosen based on current recommendations in the veterinary or human literature. Therefore, the results of this study could reflect inappropriate drug levels. Further studies involving oral and intravenous administration of these different drugs at different dosages are warranted. The method of EMG recordings could also affect the results; a system composed of multiple electrodes may have been more appropriate to detect the electrical signal when no drug was present. Finally, the clinical status and breed of dog used in this study may not be representative of the wider canine population as these animals were all healthy female dogs without any signs of urinary incontinence and with no predisposition to developing micturition disorders.

## Conclusions

Telemetry appears to be a useful investigative tool to obtain physiological data on bladder function in dogs. The results of this pilot study suggest that circadian variations may influence urodynamic measurements and that long-term telemetric studies of the lower urinary tract should be conducted during the night to obtain repeatable recordings. The investigation of urethral smooth muscle function by EMG needle electrodes appears to be a feasible method to study the effects of drugs with urethral tropism. Further pharmacokinetic and pharmacodynamics studies are needed to confirm the effects of the different drugs on vesico-urethral function.

## Methods

This study was approved by the Institutional Animal Ethical Committee of the University of Liège (protocol agreement #1084).

### Dogs

Six adult entire female Beagle dogs were included in the study. All dogs were healthy, in anoestrus and free of lower urinary tract disease. The mean age of the dogs was 53.3 ± 17.1 months old (mean ± SD) and the mean weight was 15.9 ± 1.5 kg (mean ± SD). They were housed at the animal facilities of the Research Unit of the Department of Clinical Sciences of the College of Veterinary Medicine, University of Liège. Animal housing, care, and experimentation were in accordance with Belgian governmental regulations and with the NIH Guide for Care and Use of Laboratory Animals.

### Part 1: Diuresis cystometry

Diuresis cystometry was performed in all 6 dogs under general anaesthesia induced with an IV bolus of propofol (Ecuphar, Belgium, 6 mg/kg) and maintained with an IV infusion of propofol at a maximal dosage of 0.3 mg/kg/min in order to maintain a light and stable depth of anaesthesia without need of tracheal intubation. Furosemide (Intervet, Belgium, 5 mg/kg, IV) was administrated prior to starting the IV administration of Lactated Ringer’s solution (20 mL/kg/h). Dogs were placed in right lateral recumbency and a 10-F triple lumen perfusion catheter was inserted into the bladder via the external urethral meatus and positioned such that the urethral side-hole was placed at the level of maximal urethral pressure. Bladder and urethral pressures were recorded until micturition was detected (Libra + Medical Measurement Systems, Benetec, Belgium). At that moment, fluid was retrieved from the bladder to measure the threshold volume, and anaesthesia was discontinued [5].

### Part 2: Urodynamic telemetric investigation

Telemetry transmitters (Physio Tel Multiplus transmitter TL11M3-D70-PCTP, DSI Data Science International, USA) are designed to measure combinations of pressure, biopotentials, temperature and activity. Transmitters are composed of 2 biopotential leads and 2 fluid-filled catheters with a thin-walled tip covered with an antithrombogenic film and filled with noncompressable fluid. Sensitive pressure changes are detected in the thin-walled section of the catheter and transferred to the pressure sensor located in the transmitter body. Recordings are digitized in the electronic module and data are wirelessly sent via radio frequency waves to a remote receiver (RMC-1 Physio Tel, DSI Data Science International, USA) placed in the lateral wall of a metabolic cage with a maximal transmission distance of 1 cubic meter. Signals are converted inside the data exchange matrix (Dataquest ART Data Exchange Matrix, DSI Data Science International, Sa USA). Data are transferred to a computer by an acquisition and analysis system (Dataquest Acquisition and Analysis System ART, DQ ART 3.1 Gold CM, DSI Data Science International, USA).

#### Surgical implantation

Transmitters were aseptically placed in all 6 dogs after premedication with medetomidine (Eurovet, Nederlands, 15 μg/kg, IV). Anaesthesia was induced with propofol (3 mg/kg, IV) and maintained with isoflurane (Ecuphar, Belgium, 0.8–2%) delivered in 100% oxygen using a rebreathing system. Dogs also received carprofen (Pfizer, Belgium, 4 mg/kg, IV) and cefazolin (Sandoz, Belgium, 20 mg/kg, IV).

A ventral midline celiotomy was performed and the transmitter body was secured subcutaneously to the left abdominal wall. The leads of the transmitter were passed into the abdominal cavity through the abdominal wall. The extremity of the first pressure catheter was placed into the bladder through a hole made in the ventral bladder wall and was secured to the bladder wall with a purse-string suture. The second pressure catheter was left free in a caudal intraabdominal position. The 2 EMG leads were placed into the smooth muscle part of the urethral wall (confirmed by histology of a biopsy specimen) at the level of the cranial third of the urethra, and secured with simple interrupted sutures. The abdomen was routinely closed. Postoperatively, the dogs received buprenorphine (Ecuphar, Belgium, 15 μg/kg, IV, QID) for 48 hours, carprofen (Pfizer, Belgium, 2 mg/kg, PO, BID) for 4 days, and cefazolin (Intervet, Belgium, 20 mg/kg, PO, BID) for 6 days.

#### Study protocol

Telemetric recording started one month after surgical implantation to decrease the risk of detrusor instability secondary to surgery. Dogs were placed in a metabolic cage with a uroflowmetry system for urine collection. Prior to recording, each dog had a period of acclimatisation to the cage. During this period, completion of micturition was confirmed by abdominal ultrasonography. The telemetric study was divided into 2 phases: the first phase included a day study and a night study, and the second phase included the study of the 5 urological drugs. The same 6 dogs were included in both phases.

#### First phase

To study the influence of circadian rhythym on vesico-urethral function, the dogs were placed in the metabolic cage in a lighted room and telemetric recordings were performed continuously from 7 am to 7 pm for 8 days. Upon completion of the day study, the dogs were placed in the metabolic cage in a dark room and telemetric recordings were performed continuously from 7 pm to 7 am for 8 nights.

#### Second phase

The immediate-release form of each drug was administered orally for 15 days. Telemetric recordings were performed in a dark room over night for 12 hours on day 0 (before administration), and then on days 1, 8 and 15. A two-week washout period was respected between each drug study.

The following doses of drugs were used: 1.5 mg/kg SID for PPA (Vetoquinol, Belgium), 1 mg/dog SID for oestriol (Intervet, Belgium), 12.5 mg/dog TID for bethanechol (Merck Frosst, Canada), 3.75 mg/dog BID for oxybutynin (Sanofi-Aventis, Belgium), and 20 mg/dog BID for duloxetine (Eli Lilly, Netherlands).

Telemetric data were sampled at a frequency rate of 500 Hz and a moving average of 1 second was applied. EMG data were sampled at 250 Hz. During telemetric recordings, the activity of the dogs was continuously recorded by a surveillance camera (IP/Analog Surveillance camera, Digitus, Duitsland). This camera is a network-based digital surveillance device with a built-in web server for the purpose of remote monitoring and recording. Video surveillance of the dogs over IP network infrastructure was available at all times, on the author’s computer. This closed-circuit camera system was set up and synchronised with the telemetric program. This enabled differentiation between micturition and IDC as only micturition was associated with urine expulsion.

#### Data interpretation

The following variables were measured by diuresis cystometry: threshold pressure (Pth, intravesical pressure at the time of micturition reflex), threshold volume (Vth, volume of fluid retrieved from the urinary bladder at micturition reflex), and compliance (C). Compliance was calculated by use of the following equation: C = (Vth – V0)/ (Pth – P0), where V0 and P0 are the intravesical volume and pressure, at the start of cystometric evaluation. These definitions are in accordance with those of the International Continence Society [14].

The following variables were measured or calculated from continuous telemetric recordings of the filling phases: abdominal pressure, bladder pressure and detrusor pressure (Detrusor P: bladder pressure – abdominal pressure). The data were averaged over 15 minutes periods.

The following variables were measured or calculated from telemetric recordings taken at the time of the micturition reflex: threshold bladder pressure (Pth), voided volume (Vv) and threshold abdominal pressure. The threshold detrusor pressure (Pdet th) was calculated by use of the following equation: Pdet th = Pth – Pabd th.

**Figure 2:**
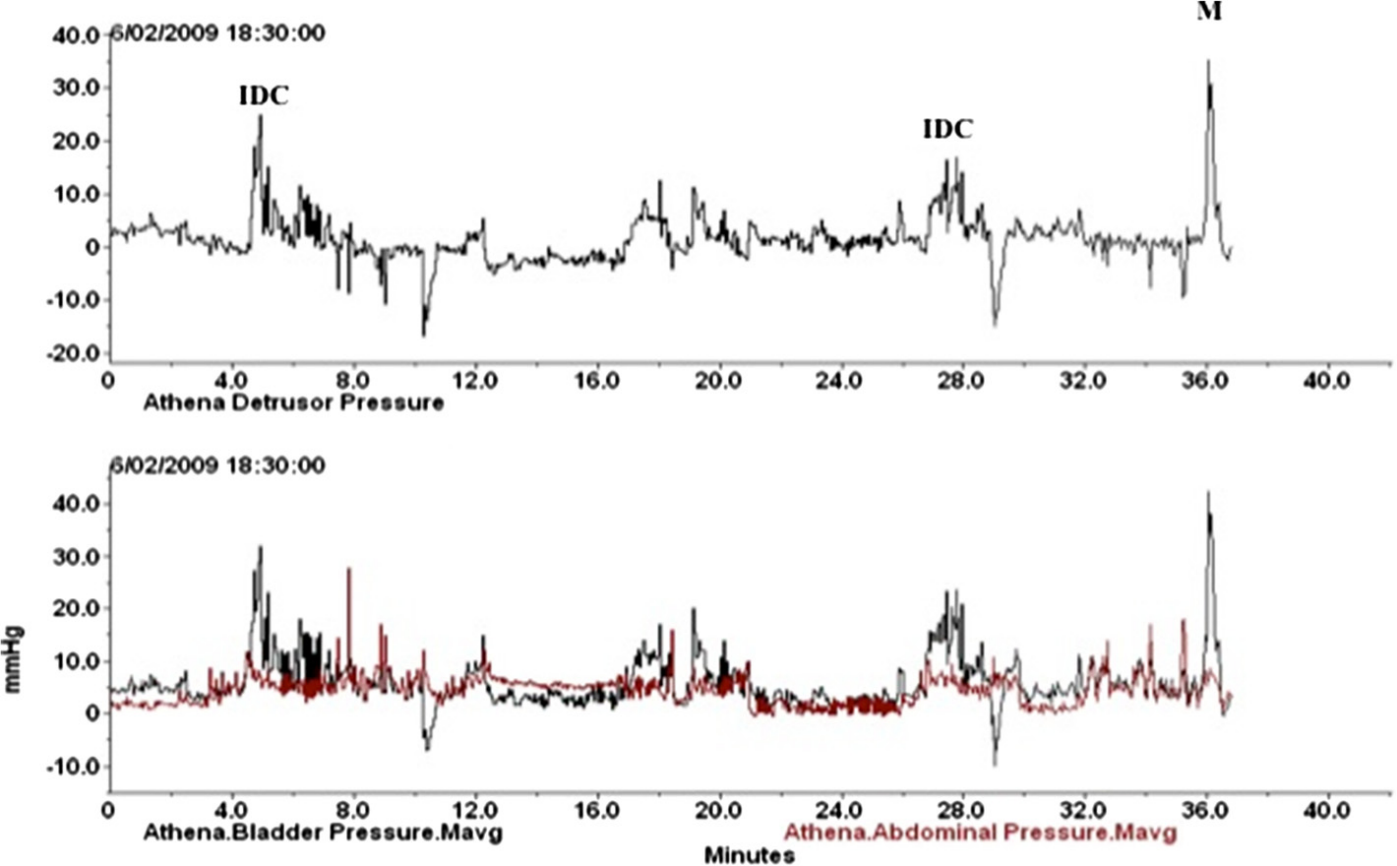
**Telemetric recording.** IDC = Involuntary detrusor contraction, M = Micturition.

IDC were recorded throughout telemetric recordings and were defined as a rapid involuntary pressure increase of ≥ 15 cmH_2_O occurring during the filling phase at a lower threshold volume than would be expected to provoke a normal micturition reflex (Figure 2) [76].

The electrical activity of the urethral smooth muscle was studied during the hour around each micturition episode. EMG signals were filtered from 2 to 40 Hz and were analysed by calculating the EMG integral activity (EMGi) over 2 seconds. EMGi values were obtained 30 minutes, 5 minutes, 1 minute and 10 seconds before the micturition episode, at the time of micturition, and 10 seconds, 1 minute, 5 minutes and 30 minutes after the end of micturition. The global smooth muscle activity during the hour around the micturition episode (EMGi/hour) was measured by taking the mean of these different EMGi values.

The electrical activity of the urethral smooth muscle was also analysed during the minute immediately preceding the micturition episode and immediately following the micturition episode. The EMGi value (EMGi/minute) was calculated over 60 seconds.

#### Statistical analysis

Data were expressed as Least Square Mean (LSMean) ± SEM. Urodynamic data obtained by diuresis cystometry and telemetric investigation were analysed and compared using the GLM procedure (SAS/STAT software, version SAS (1) 9.1 (TS1M3) on Linux 2.4.21-50, ELsmp platform, SAS Institute Inc, Cary, NC). The frequency of micturition during the first phase of the telemetric study was analysed and compared by the LOGISTIC procedure of the SAS software, in agreement with the discreet character of this variable. The MIXED procedure of the SAS software was used to analyse and compare the urodynamic and electromyographic data obtained during the day and night studies, as well as during the administration of PPA, oestriol, bethanechol, oxybutynin and duloxetine. These statistical models take into account the possible correlations between successive measures over time. A value of *P* < 0.05 was considered significant.

## Abbreviations

EMG: Electromyography; EMGi: EMG integral activity; IDC: Involuntary detrusor contraction; LSMean: Least Square Mean; PPA: Phenylpropanolamine; Pth: Threshold bladder pressure; Pdet th: Threshold detrusor pressure; SUI: Stress urinary incontinence; USMI: Urethral sphincter mechanism incompetence; Vv: Voided volume.

## Competing interests

The authors declare that they have no conflicting interests.

## Authors’ contributions

SN participated in the conception of the study and study design and wrote the manuscript. LM performed the statistical analysis. AH conceived the study and reviewed and corrected the manuscript. All authors read and approved the final manuscript.
